# Detection of *B. anthracis* Spores and Vegetative Cells with the Same Monoclonal Antibodies

**DOI:** 10.1371/journal.pone.0007810

**Published:** 2009-11-13

**Authors:** Dian-Bing Wang, Ruifu Yang, Zhi-Ping Zhang, Li-Jun Bi, Xiang-Yu You, Hong-Ping Wei, Ya-Feng Zhou, Ziniu Yu, Xian-En Zhang

**Affiliations:** 1 State Key Laboratory of Virology, Wuhan Institute of Virology, Chinese Academy of Sciences, Wuhan, China; 2 State Key Laboratory of Agromicrobiology, College of Life Science and Technology, Huazhong Agricultural University, Wuhan, China; 3 State Key Laboratory of Macromolecules, Institute of Biophysics, Chinese Academy of Sciences, Beijing, China; 4 State Key Laboratory of Pathogen and Biosecurity, Institute of Microbiology and Epidemiology, Academy of Military Medical Sciences, Beijing, China; Tsinghua University, China

## Abstract

*Bacillus anthracis*, the causative agent of anthrax disease, could be used as a biothreat reagent. It is vital to develop a rapid, convenient method to detect *B. anthracis*. In the current study, three high affinity and specificity monoclonal antibodies (mAbs, designated 8G3, 10C6 and 12F6) have been obtained using fully washed *B. anthracis* spores as an immunogen. These mAbs, confirmed to direct against EA1 protein, can recognize the surface of *B. anthracis* spores and intact vegetative cells with high affinity and species-specificity. EA1 has been well known as a major S-layer component of *B. anthracis* vegetative cells, and it also persistently exists in the spore preparations and bind tightly to the spore surfaces even after rigorous washing. Therefore, these mAbs can be used to build a new and rapid immunoassay for detection of both life forms of *B. anthracis*, either vegetative cells or spores.

## Introduction

*Bacillus anthracis*, the causative agent of anthrax disease, is a Gram-positive spore-forming bacterium. Fully virulent bacilli carry two plasmids, pXO1 and pXO2, which contain genes to produce the lethal factor and edema factor toxins, and a poly-γ-D-glutamic acid capsule, respectively [1], [2]. In response to nutrient deprivation, *B. anthracis* will produce spores that can withstand harsh conditions, including temperature, radiation, chemical assault, time and even the vacuum of outer space [3]. These remarkable characteristics allow *B. anthracis* to be used as a biological threat agent.

Recent studies have indicated that *B. anthracis* is genetically similar to other members of the *Bacillus* genus. The 16S rRNA, 23S rRNA and 16S–23S internal transcribed spacer sequences of *B. anthracis* share a high degree of similarity with those in *B. cereus*, *B. subtilis*, *B. megaterium*, *B. mycoides*, and *B. thuringiensis* [4], [5]. These sequence similarities make identification of *B. anthracis* challenging, and substantial effort has been devoted to developing identification methods. The conventional method is the bacteriological assay, which is reliable but time consuming. Other advanced approaches have been proposed for *B. anthracis* detection, including immunological assays [6], [7], PCR-based methods [8], [9], [10], [11], [12], [13], molecular fingerprinting [14], [15], [16] and mass spectrometric (MS) analyses [17], [18]. The detection targets have mainly focused on the pXO1 and pXO2, gene polymorphisms, specific gene sequences (*Ba813*, *rpoB, bclA*) and small acid soluble proteins (SASPs) in the spores. However, these methods fail to eliminate the need for complicated protocols such as cell disruption, nucleic acid extraction and protein purification, and cannot support convenient, rapid and real-time detection of *B. anthracis*. Therefore, direct detection of *B. anthracis* is attractive for on-site application. So far, several immunoreagents, directed against the surface of *B. anthracis*, have been developed for the direct detection of *B. anthracis* vegetative cells or *B. anthracis* spores [19], [20], [21], [22], [23].

In the current study, attempts were also made to generate mAbs using fully washed *B. anthracis* spores as an immunogen. Unexpectedly, we identified three high affinity mAbs (8G3, 10C6 and 12F6) capable of direct and species-specific recognition of both *B. anthracis* spores and vegetative cells. Furthermore, these mAbs were all directed against the EA1 protein, which is well known as a major S-Layer protein in *B. anthracis* vegetative cells [24], [25]. Some recent studies have revealed that EA1 is also retained in the proteomic profiling of rigorous washed spores and even salt/detergent washed exosporium [26], [27]. Although Williams and Turnbough suggested EA1 was not a true spore surface protein, they stated this protein persistently existed in the spore surface [28]. Therefore, it is valid to use EA1 as a detection target of *B. anthracis* spores herein, as EA1 is highly associated with the spore surface. In this study, we conclude that the mAbs we prepared, directed against EA1, can recognize the surface of *B. anthracis* spores as well as vegetative cells, and we also suggest EA1 protein can serve as a potential marker for the detection of *B. anthracis*. This study is significant because until now, there have been no monoclonal antibodies reported for direct and species-specific detection of both life forms of *B. anthracis*.

## Results

### Atomic-force microscopy (AFM) analysis of *B. anthracis* spores

**Figure 1:**
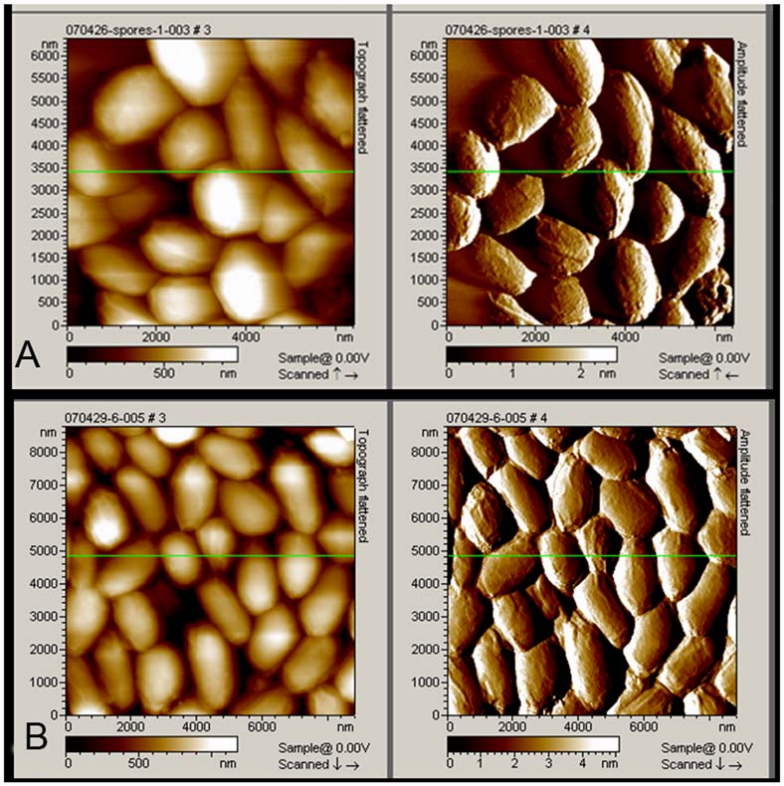
Images captured by AFM analysis of *B. anthracis* spores. Topography images (left) and amplitude images (right) of the spores were collected in tapping mode. A: Unwashed spores. B: Extensively washed spores.

To guarantee the purity of spores that we prepared, both unwashed and fully washed spores were analysed by AFM. As is shown in Figure 1, a larger amount of free spores appeared. Even the unwashed spores were surprisingly clean, as no intact vegetative cells and little vegetative cell debris were present (Fig. 1A). However, because it is likely that some vegetative cell proteins will bind accidentally to the spore surface, the rigorous washing method, as described above, was still employed. There were few differences between the unwashed spores and fully washed spores, but the latter seemed to have a much smoother surface than the former, indicating that our spore purification protocol had removed some unknown material (Fig. 1B). Therefore, we used these fully washed *B. anthracis* spores as our immunogen and employed them in the subsequent experiments.

### Preparation and screening of mAbs against *B. anthracis* spores

**Figure 2:**
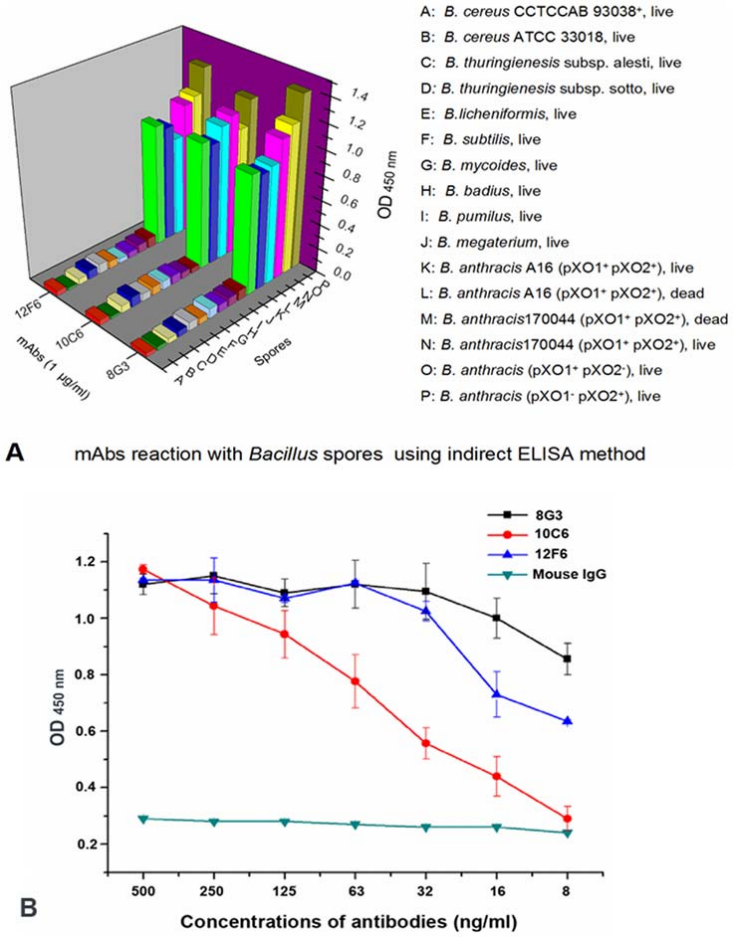
The specificity and reactivity of mAbs with *B. anthracis* spores. A: Reaction of the mAbs with a series of *Bacillus* spores, as detected by indirect ELISA. B: Reactions of different concentrations of mAbs with *B. anthracis* spores by indirect ELISA. The experiment was repeated three times.

By the fusion protocol [29], approximately 600 hybridoma cultures were screened with the indirect ELISA method. Fifteen hybridomas produced high affinity antibodies and were cloned successfully. Following isotyping, five mAbs were IgMs, while the other 10 were IgGs (data not shown). These mAbs were then examined for their reactivity against a range of *Bacillus* spores using ELISAs. Three mAbs, designated 8G3 (IgG3), 10C6 (IgG1) and 12F6 (IgG1), specifically recognized *B. anthracis* spores (Fig. 2A), and showed no cross-reaction with high concentrations of *B. cereus*, *B. subtilis*, *B. megaterium*, *B. mycoide*s and other *Bacillus* spores. Additionally, compared to the other mAbs, these three mAbs all had higher affinity against *B. anthracis* spores. Even with a range of low concentrations, these mAbs, especially mAb 8G3, always showed strong signals when reacting against *B. anthracis* spores (Fig. 2B). Therefore, these three mAbs were examined further.

### Antigen identification

**Figure 3:**
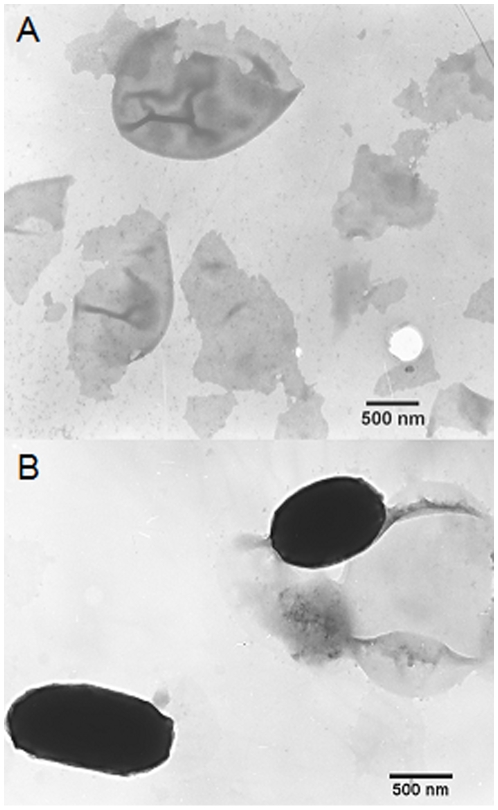
Transmission electron micrographs. A: *B. anthracis* exosporium fragments and the *B. anthracis* spores after sonication. B: Scale bars: 500 nm.

**Figure 4:**
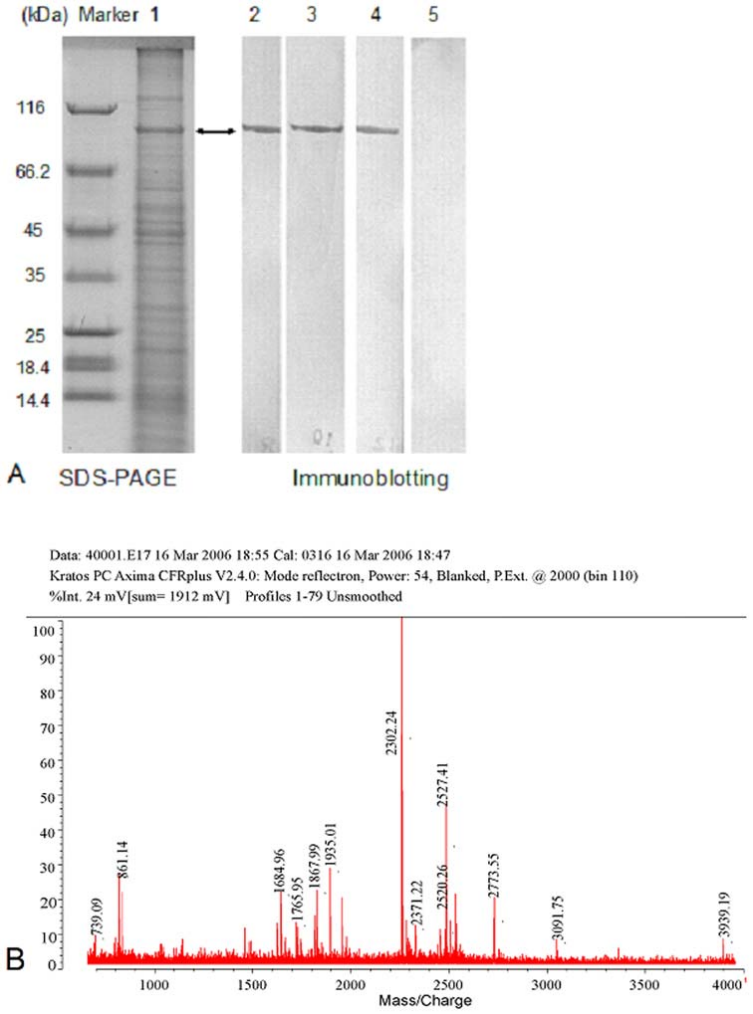
Identification of the mAbs' target proteins. A: The protein profiles of the fully washed *B. anthracis* exosporium following SDS-PAGE (Lane 1) and immunoblotting with mAb 8G3 (Lane 2), 10C6 (Lane 3), 12F6 (Lane 4) and mouse IgG (Lane 5). B: Mass spectrometric analysis of the target protein, which was determined to be EA1.

**Figure 5:**
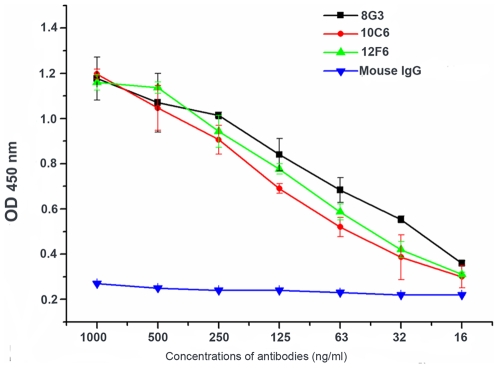
The reactivity of the mAbs with purified EA1 protein. Reactions of a range of concentrations of the mAbs with the 4 µg/ml EA1 protein using indirect ELISA. The experiment was repeated three times.

Exosporium, the outermost structure of spores, was isolated from *B. anthracis* A16 spores using mild sonication in order to avoid the loss of some exosporium proteins and the damage of the remaining spore protein denaturation. Using electron microscopy, it was observed that the exosporium fragments were successfully extracted (Fig. 3A), and there was no obvious disruption to the spores after sonication (Fig. 3B). The exosporium fragments were boiled in sample buffer for 30–40 min to release more soluble proteins. The soluble exosporium proteins were separated by SDS-PAGE, and the mAbs (8G3, 10C6 and 12F6) recognized a 91–93 kDa protein by Western blotting (Fig. 4A). The band was excised and subjected to DE-MALDI-TOF-MS analysis; the protein was identified as EA1 (Fig. 4B). To determine whether the mAbs were all directed against EA1, the coding region of the gene for EA1 was cloned into the pQE-30 plasmid and expressed in *E. coli* M15, and the expressed proteins were purified with affinity chromatography. By the method reported previously [30], the affinity constants (*K_aff_*) of the mAbs (8G3, 10C6 and 12F6) for the recombinant EA1 protein were measured to be 1 to 3×10^9^ M^−1^ Thus, the results not only established that EA1 was the target protein of the three mAbs, but also indicated that the mAbs had a high affinity for EA1.The reaction curves for the mAbs with 4 µg ml^−1^ recombinant EA1 are shown in Figure 5.

### The mAbs could also react with *B. anthracis* vegetative cells

Since EA1 is a major S-Layer protein in *B. anthracis* vegetative cells [24], [31], the mAbs against EA1 were tested for their reactivity and specificity to *B. anthracis* vegetative cells. The sandwich ELISA method was employed, rather than indirect ELISA, because the intact vegetative cells were so large that were not adequately coated to the microwells in our indirect ELISA assays (data not shown).

**Figure 6:**
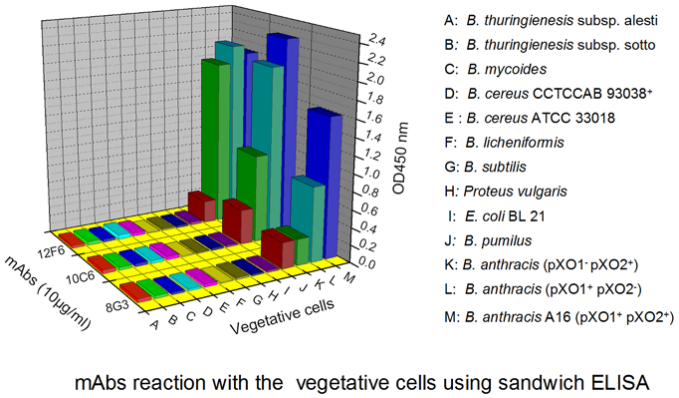
The specificity and reactivity of the mAbs with *B. anthracis* vegetative cells. Reactions of the mAbs with a series of vegetative cells, as detected by sandwich ELISA.

As shown in Figure 6, the mAbs specifically recognized *B. anthracis* vegetative cells with no significant cross-reaction with other strains at high concentrations (10^7^ CFU/ml). Of these mAbs, 12F6 seemed the best candidate, because it reacted strongly with different kinds of *B. anthracis* vegetative cells. However, the other mAbs, especially 8G3, did not strongly recognize *B. anthracis* strains lacking the pXO1 or pXO2 plasmids. Therefore, these results indicated that the mAbs had different characteristics, despite having been raised against the same protein.

### The mAbs bound to surfaces of both *B. anthracis* spores and vegetative cells

**Figure 7:**
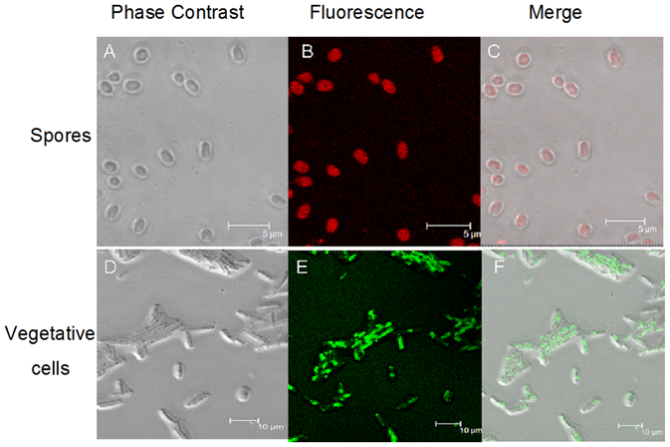
Representative confocal microscopy images of 8G3 binding to fully washed *B. anthracis* spores (top) and vegetative cells (bottom). The secondary antibodies were R-phycoerythrin-conjugated goat anti-mouse IgG. (red) or FITC-conjugated goat anti-mouse IgG (green). The corresponding phase contrast images (A and D) and fluorescence images (B and E) were merged to show overlap (C and F). Scale bars: spores, 5 µm; vegetative cells, 10 µm.

Binding of mAbs (8G3, 10C6 and 12F6) to the surface of *B. anthracis* A16 spores and vegetative cells was investigated with immunofluorescence. Normal mouse IgG was used as a negative control (Figure S1), and photographs from the 8G3 experiments as examples are shown in Figure 7. In the immunofluorescence assays, the fully washed spores incubated with 8G3 were extensively stained with R-phycoerythrin-conjugated goat anti-mouse IgG (red). The vegetative cells were incubated with the mAb, and were visualized with FITC-conjugated goat anti-mouse IgG (green). The pattern of fluorescence indicated that these mAbs not only recognized the surfaces of spores, but also bound to the intact vegetative cells.

### EA1 was persistently present in *B. anthracis* spore extracts

During preparation, the *B. anthracis* spores were washed extensively with water and pelleted through 20% and 50% Renografin to remove vegetative cell debris [10]. The spores were monitored and collected at four stages of the standard washing procedure: unwashed spores from the culture plate (Preparation 1), spores washed with MilliQ water for 24 h, three times (Preparation 2), Renografin purified spores (Preparation 3), and spores subjected to three additional washes (Preparation 4). In addition, the supernatants from sedimentation during Preparation 3 were diluted 1∶10 with water and centrifuged at 10,000×g for 30 min to collect the debris. Equal volumes of all preparations and the debris were subjected to SDS-PAGE analysis.

**Figure 8:**
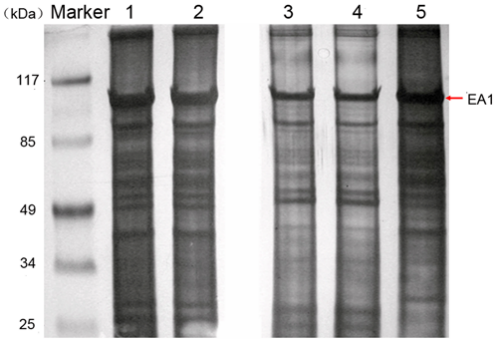
Comparison of the protein profiles of spores and debris. Proteins were separated on a 10% Tricine gel and visualized with silver staining. Lane 1, unwashed spores (Preparation 1); Lane 2, spores washed three times with ultrapure water (Preparation 2); Lane 3, spores prepared by Renografin purification (Preparation 3); Lane 4, fully washed spores (Preparation 4); Lane 5, debris in the supernatant of centrifuged spores after Renografin purification. (The staining time of Preparation 3, 4 and 5 was longer than that of Preparation 1 and 2, in order that more bands of each sample could be visualized clearly).

As a result, the *B. anthracis* A16 SDS-PAGE profiles showed no obvious differences between Preparations 1 and 2, except that a negligible amount of protein was removed by the washing steps (Figure 8). The same results were observed when Preparations 3 and 4 were compared. In contrast, most proteins, including EA1, decreased after sedimentation through Renografin. These removed proteins were present in the “debris” of the supernatant, which contained a similar amount of protein to Preparations 2 and 4. These results suggest that the Renografin step washed out some of the EA1 along with many other spore proteins, which were then detected by SDS-PAGE. Despite the presence of EA1 in the debris, EA1 was also persistently present in the *B. anthracis* A16 spore extracts of each preparation. Furthermore, *B. anthracis* strains lacking pXO1 or pXO2 produced similar results to those presented above (data not shown). Therefore, EA1 was persistently present in *B. anthracis* spore extracts.

## Discussion

The primary goal of this study was to generate mAbs with high affinity and specificity that could be applied to rapid detection of *B. anthracis* spores. The mAbs were produced against formaldehyde-inactivated *B. anthracis* A16 spores and reacted with a range of live *Bacillus* spores, including *B. anthracis.* Most of the mAbs we produced were highly specific for *B. anthracis* spores. For each screening of the hybridoma cultures, spores from *B. cereus* and *B. thuringiensis*, the two closest relatives of *B. anthracis*, were used as negative controls. To identify mAbs with high affinity and specificity, hybridomas were selected if the mAbs reacted strongly with *B. anthracis* but did not recognize either negative antigen. As a result, the three mAbs (8G3, 10C6 and 12F6) we prepared have no cross reaction with many *B. thuringienesis* subspecies and *B. cereus* isolates (Table S1).

The three mAbs recognized not only the surface of *B. anthracis* spores but could also detect intact *B. anthracis* vegetative cells (Fig. 7). Furthermore, these mAbs were capable of reaction with live *B. anthracis* as well as dead *B. anthracis* (inactivated by 1.5% formaldehyde), which is critical for the detection of biological warfare agents in unknown “white powders”, since it has been suggested that *Bacillus* inactivation would affect antibody detection assays [32]. Although these three mAbs were directed toward the same target protein, EA1, they had different characteristics. The mAb 12F6 was superior at reacting with different kinds of *B. anthracis* vegetative cells, while 8G3 had a higher affinity for *B. anthracis* spores and the target protein EA1 (Fig. 2, Fig. 5 and Fig. 6). Besides this, in the epitope mapping, the epitopes of mAb 8G3 and 10C6 were concluded to be located from the amino acid 275 to 435 on the EA1 protein, and the epitope of mAb 12F6 was exactly located from the amino acid 465 to 554 (data not shown). We suggested that the different positions of the mAb epitopes caused the mAbs to exhibit different behavior the detection of *B. anthracis*.

As to whether EA1, a major S-layer component of *B. anthracis* vegetative cells, is also a spore protein, much research has indicated that this protein is retained in the proteomic profiling of spores and salt/detergent washed exosporium [26], [27]. However, Williams and Turnbough stated that this protein was merely a persistent contaminant in spore preparations [28]. Whichever is correct, it does not matter for the detection of *B. anthracis* spores, because this protein does persistently exist in each of the spore preparations (Fig. 8), and the *B. anthracis* spores, even after full washing, can be detected with our anti-EA1 mAbs (Fig. 2 and Fig. 7).

Although EA1 could be partially washed out during the rigorous washing step in our study, the other proteins detected by SDS-PAGE also apparently decreased and were present as debris in the supernatant of centrifuged purified samples (Fig. 8). The supernatant debris contained significant amounts of true spore proteins with similar protein profiles to fully washed spores. Therefore, rigorous washing methods, such as Renografin purification, can cause serious loss of spore surface associated proteins and are not suggested for proteomic analysis [26], [27]. This suggests that EA1 is certainly at least a highly spore-associated protein: it might be a true spore protein, or may anchor onto particular spore surface components.

In conclusion, this study reports three mAbs (8G3, 10C6 and 12F6) that can bind to *B. anthracis* spores and intact vegetative cells with high species-specificity and affinity. It also indicates that EA1, the target protein of our mAbs, could serve as a potential detection target of *B. anthracis*, establishing a new immunoassay protocol that realizes sensitive, rapid, on-site and simultaneous detection of both life forms of *B. anthracis*.

## Materials and Methods

### Bacterial strains

The bacterial strains used in this study are listed in Table 1. All assays involving live *B. anthracis* spores and vegetative cells were carried out in a P3 biosafe laboratory. The experimenters were equipped with masks, gloves and exposure suits.

**Table 1:** Bacterial strains examined in this study.

Strains	Source	Plasmid	
		pXO1	pXO2
*B. anthracis* A16	Institute of Microbiology and Epidemiology, AMMS	+	+
*B. anthracis* 170044	Institute of Microbiology and Epidemiology, AMMS	+	+
*B. anthracis* CMCC(B) 63002	National Center for Medical Culture Collections, China	+	−
*B. anthracis* CMCC(B) 63005	National Center for Medical Culture Collections, China	−	+
*B. cereus* CCTCCAB 93038+	Institute of Microbiology and Epidemiology, AMMS		
*B. cereus* ATCC 33018	Huazhong Agricultural University, China		
*B. thuringienesis* subsp. alesti	Huazhong Agricultural University, China		
*B. thuringienesis* subsp. sotto	Huazhong Agricultural University, China		
*B.licheniformis*	Institute of Microbiology and Epidemiology, AMMS		
*B. subtilis*	Institute of Microbiology and Epidemiology, AMMS		
*B. megaterium*	Institute of Microbiology and Epidemiology, AMMS		
*B. mycoides*	Wuhan Institute of Virology, Chinese Academy of Sciences		
*B. pumilus*	Wuhan Institute of Virology, Chinese Academy of Sciences		
*Proteus vulgaris*	Wuhan Institute of Virology, Chinese Academy of Sciences		
*E. coli* BL21	Wuhan Institute of Virology, Chinese Academy of Sciences		
*E. coli* M15	Wuhan Institute of Virology, Chinese Academy of Sciences		

AMSS: the Academy of Military Medical Sciences, China.

### Preparation of spores

Spores were prepared by growing bacteria at 37°C on modified Difco sporulation medium (DSM) [33], containing 6 g tryptone, 3 g yeast extract, 10 g NaCl, 1 g KCl, 0.25 g Mg_2_SO_4_·7H_2_O, 0.23 g Ca(NO_3_)_2_, 0.197 g MnCl_2_·4H_2_O, 0.0002 g FeSO_4_, and 15 g agar per liter. When more than 95% free spores appeared, the spores were collected with cold, sterile, ultrapure water, centrifuged and washed extensively three times, before sedimentation through 20% and 50% Renografin (Bracco Sine, China) [10] and another three washes. The washed spores were then resuspended in sterile saline and stored at 4°C. The spores were quantified by serially diluting the spore stock, and plating 100 µl aliquots on Luria broth (LB) plates, in triplicate. The number of spores (CFU/ml) was counted following overnight culture at 37°C.

### Atomic-force microscopy (AFM) analysis of spores

An aqueous spore suspension of 10 µl, containing 10^8^–10^9^ spores, was spread on a 1 cm×1 cm silicon wafer and air-dried. The sample was not placed in the AFM chamber for imaging until the spores settled on the substrate. Picoscan™ 2500 AFM (Molecular Imaging, US) and commercial single MAClever type II cantilevers (Molecular Imaging, US) were used to obtain images in tapping mode.

### Exosporium extraction

Approximately 0.5 g of purified *B. anthracis* spores (wet weight) were collected by centrifugation at 9,000×g for 5 min at 4°C and resuspended in 15 ml cold sterile TE buffer (50 mmol l^−1^ Tris-HCl, 0.5 mmol l^−1^ EDTA, pH 7.2), containing 1 µl ml^−1^ protease inhibitor cocktail (Sigma, US). All subsequent centrifugations were performed at 4°C. To extract larger exosporium fragments, the spore suspension was divided into three partitions and each was treated using a Vibra-Cell Ultrasonic Processor (Sonics & Materials, USA) with 120 pulse cycles on ice (5 s pulse - 9 s interval - 5 s pulse). The three partitions were sonicated, pooled and centrifuged for 45 min at 1,200×g. Then the pellet was washed five times with cold TE buffer and centrifuged as above. All the supernatant samples were saved and centrifuged at 184,000×g for 1 h. The sediments, which were composed of exosporium fragments, were then resuspended in 500 µl sterile PBS for further study.

### Production of monoclonal antibodies and polyclonal antibodies

Preparations containing 10^6^ spores of *B. anthracis* strain A16 and inactivated by 1.5% formaldehyde were injected subcutaneously into six-week-old BALB/c mice. The immunization was repeated three times at two-week intervals before boosting by intraperitoneal injection. The spleen cells were removed 3 d later and fused with SP2/0 myeloma cells, according to the procedures of Kohler and Milstein (1975)[29]. The hybridomas were cloned by limit dilution, screened using ELISA (described below) and then injected intraperitoneally into BALB/c mice. The mAbs were isotyped using an isotyping kit (Sigma, US), according to the manufacturer's directions, and were purified by caprylic acid-ammonium sulfate precipitation of ascites [34].

For the polyclonal antibodies against *anthracis* vegetative cells, the rabbit was injected subcutaneously with 10^7^ cells (inactivated by 1.5% formaldehyde) for three times at two week before boosting by double injection dose. The polyclonal antibodies were purified from the antiserum by caprylic acid-ammonium sulfate precipitation of ascites.

### ELISA

For indirect ELISA detection, 96 well microtiter plates were coated with 100 µl per well of carbonate-bicarbonate (CB) buffer (pH 9.6) containing either 10^7^–10^8^/ml spores or recombinant EA1 (2, 4, 8, 16 µg/ml) and incubated overnight at 4°C. The wells were blocked with 200 µl of blocking buffer (5% skim milk in phosphate buffered saline (PBS) for 2 h at 37°C. Hybridoma culture supernatants or a concentration series of purified mAbs were added in 100 µl aliquots to individual wells and incubated for 30 min at 37°C. Horseradish peroxidase (HRP)-conjugated goat anti-mouse antibodies were added at a dilution of 1/900 and incubated for 20 min.

For sandwich ELISA, the microtiter plates were coated with purified mAbs (10 µg/ml) and blocked as described above. The plates were incubated with a range of *Bacillus* vegetative cells at 37°C for 1 h and then reacted with 10 µg/ml polyclonal antibodies against anthrax at 37°C for 40 min. HRP-conjugated goat anti-rabbit antibodies were added to the plate at a dilution of 1/3000 (Boster, China) and reacted for 30 min at 37°C.

For both indirect ELISA and sandwich ELISA, five washes with PBS with 0.05% Tween 20 (PBST) were carried out between each step. Normal mouse IgG was used as a negative control, and all antibody dilutions were prepared in PBS, containing 1% skim milk. A tetramethylbenzidine substrate (0.1 mg/ml 100 µl per well) was added for approximately 10–15 min at 37°C to start the reaction, and 50 µl of 2 M H_2_SO_4_ was added to stop the reaction. Then the absorbance was measured at 450 nm, and each assay was performed in quadruplicate.

### Analysis by SDS-PAGE and immunoblotting

Samples were mixed with an equal volume of sample buffer (50 mM Tris-HCl at pH 6.8, 0.5 mol l^−1^ β-mercaptoethanol, 20% v/v glycerol, 10% w/v SDS, 0.2% w/v bromophenol blue) and boiled for 10–40 min. The soluble proteins were analyzed by SDS-PAGE using a Bio-Rad gel apparatus, according to the manufacturer's instructions. For immunological detection, the proteins were transferred from the gels onto polyvinylidene difluoride (PVDF) membranes (Millipore, US). The membranes were blocked in Tris-buffered saline (TBS) containing 5% skim milk at 4°C overnight, and were washed three times for 5 min with Tris-buffered saline Tween-20 (TBST, with 0.05% Tween 20). Then the membranes were incubated in 5 µg ml^−1^ mAbs, which were diluted in TBS containing 1% skim milk, for 1.5 h at room temperature, followed by three additional 10 min washes. The membranes were incubated for 1 h with a 1/100 dilution of HRP-conjugated goat anti-mouse IgG. Four thorough 10 min washes were completed before the substrate buffer (6 mg 3,3′-Diaminobenzidine (DAB) and 10 µl of 37% H_2_O_2_ in 10 ml TBS) was added and allowed to react for 3–5 min. Images were acquired using a Canon digital camera and analyzed using Adobe Photoshop software.

### MS protein analysis

The protein band identified on the SDS-PAGE gel was excised, washed, and subjected to tryptic digestion [26]. The peptides were collected and analyzed by delayed extraction-matrix assisted laser desorption ionization-time of flight mass spectrometry (DE-MALDI-TOF-MS). The MS spectra were obtained using an Axima-CFR Plus Mass Spectrometer (Shimadzu Biotech, Japan). All MS analyses were performed at the Institute of Biophysics, Chinese Academy of Sciences, China.

### Gene cloning and expression

Sequence data for the *B. anthracis* genome were obtained from the National Center for Biotechnology Information (NCBI) web site at http://www.ncbi.nlm.nih.gov. Based on the sequence information, DNA primers were designed to amplify the entire *eag* open reading frame of the *B. anthracis* A16 strain using polymerase chain reaction (PCR). The chromosomal DNA of A16 was used as the template, and the primer sequences were 5′-TATTGGATCCATGGCAAA GACTAACT-3′ and 5′-CTATAGAGCTCGTATAGATTTGGGTT A -3′. The PCR conditions were as follows: 30 cycles of 94°C for 5 min, 94°C for 45 s, 40°C for 45 s, and 72°C for 2 min for 30 cycles; and lastly 72°C for 5 min. The PCR products were digested with *BamHI* and *SacI* and were inserted into plasmid pQE-30 (Qiagen, Germany). The recombinant plasmids were transformed into *E. coli* M15 to express the cloned gene *in vitro*. Firstly, the *E. coli* M15, carrying recombinant plasmids, were incubated at 37°C at 160 rpm to an average OD_600_ of 0.5 followed by addition of IPTG to 1 mM final concentration, and were incubated for another 4 h at 30°C. Secondly, the cells were harvested by centrifugation at 3000 g for 5 min, and were sonicated by a Vibra-Cell Ultrasonic Processor (Sonics & Materials, USA) as described above. Lastly, after centrifugation at 12,000 g for 20 min, the supernatant containing recombinant EA1 protein with His tag was purified using Ni-nitrilotriacetic acid agarose (Qiagen, Germany) affinity chromatography, according to the manufacturer's instructions.

### Determination of the mAbs affinities

As the antibody affinities reported previously [30], different concentrations of recombinant EA1 (2, 4, 8, 16 µg/ml) were coated on the microwells and incubated with serial dilutions of mAbs for indirect ELISAs. The affinity constants (*K_aff_*) of the mAbs (8G3, 10C6 and 12F6) for the recombinant EA1 protein were measured using the equation: *K_aff_* = (n−1)/2(n[Ab′]t−[Ab]t), where n = [Ag]/[Ag′]. Briefly, [Ag] and [Ag′] are antigen (EA1) concentrations. [Ab′]t and [Ab]t are the measurable total antibody concentrations at the half maximum OD (OD-50) for plates coated with [Ag′] and [Ag], respectively.

### Indirect immunofluorescence

For the indirect immunofluorescence assays, 200 µl aliquots of spore or vegetative cell suspension (10^9^ CFU ml^−1^) were pelleted by centrifugation at 10,000×g for 1 min at room temperature. The spores and vegetative cells were resuspended by adding 500 µl blocking buffer and incubated for 2 h at 37°C with gentle shaking (70–100 rpm). Aliquots of mAbs (500 µl; 1 µg ml^−1^) were added and the mixture was incubated for 1 h at 37°C with shaking. R-phycoerythrin-conjugated goat anti-mouse IgG (Sigma, US) or FITC-conjugated goat anti-mouse IgG (Boster, China) was used as the secondary antibody (100 µl, 1/50 dilution), and was added to the spores or vegetative cell solution respectively. The mixture was incubated for 40 min at room temperature in the dark. Each step was followed by three 10 min washes in PBST, followed by centrifugation at 10,000×g for 1 min to remove the supernatant. The antibodies were collected in PBS containing 1% skim milk, and images were acquired using a Leica confocal microscope.

## Supporting Information

Figure S1 Confocal microscopy images of negative control antibody binding to B. anthracis. The secondary antibodies were R-phycoerythrin-conjugated goat anti-mouse IgG. (B. anthracis spores, top) and FITC-conjugated goat anti-mouse IgG (vegetative cells, bottom). Scale bars: spores, 5 µm; vegetative cells, 10 µm
(0.53 MB DOC)

Table S1 Additional B. thuringienesis subspecies and B. cereus isolates than the strains shown in the manuscript were reacted with our mAbs to further determine the mAbs species specificity. The B. cereus strains were from Wuhan Institute of Virology, Chinese Academy of Sciences. The B. thuringiensis strains were from Huazhong Agricultural University, China.
(0.07 MB DOC)
