# Supplementary figures and images for: Detection of B. anthracis Spores and Vegetative Cells with the Same Monoclonal Antibodies
Source: PLoS One. 2009 Nov 13;4(11):e7810. doi: 10.1371/journal.pone.0007810 (PMC2773009; doi:10.1371/journal.pone.0007810)

Figure S1


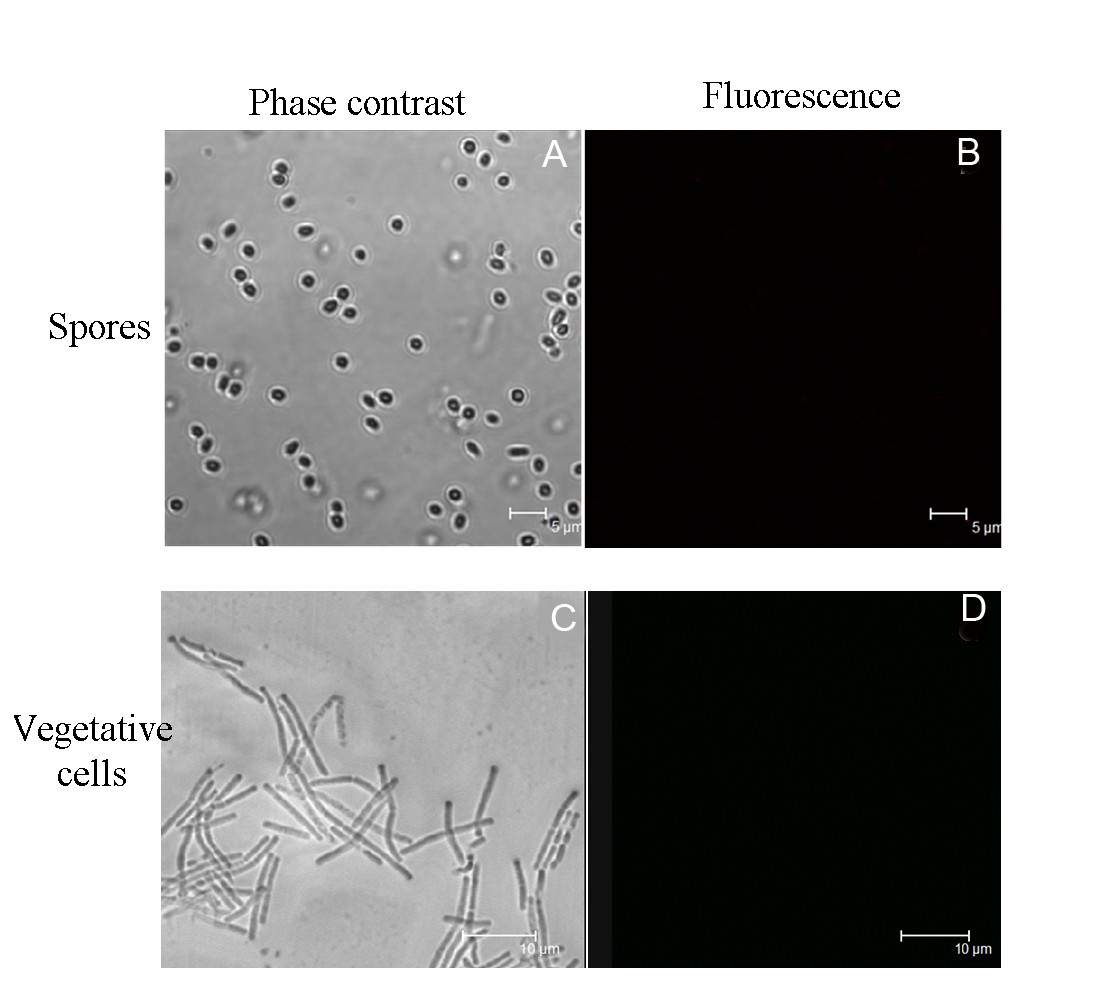

Supplement: Figure S1 — Confocal microscopy images of negative control antibody binding to B. anthracis. The secondary antibodies were R-phycoerythrin-conjugated goat anti-mouse IgG. (B. anthracis spores, top) and FITC-conjugated goat anti-mouse IgG (vegetative cells, bottom). Scale bars: spores, 5 µm; vegetative cells, 10 µm (0.53 MB DOC) [file pone.0007810.s001.doc]
