# Supplementary material for: Detection of B. anthracis Spores and Vegetative Cells with the Same Monoclonal Antibodies
Source: PLoS One. 2009 Nov 13;4(11):e7810. doi: 10.1371/journal.pone.0007810 (PMC2773009; doi:10.1371/journal.pone.0007810)
Supplement: Table S1 — Additional B. thuringienesis subspecies and B. cereus isolates than the strains shown in the manuscript were reacted with our mAbs to further determine the mAbs species specificity. The B. cereus strains were from Wuhan Institute of Virology, Chinese Academy of Sciences. The B. thuringiensis strains were from Huazhong Agricultural University, China. (0.07 MB DOC) [file pone.0007810.s002.doc]

**Table S1**

**Table 1** **mAbs reactions with the spores of *B. thuringienesis* and *B. cereus* using indirect ELISA**

| **Spores**  **(10^7^ CFU/ml)** | **OD_450 nm_** | | |
| --- | --- | --- | --- |
|  | **mAb 8G3** | **mAb 10C6** | **mAb 12F6** |
| *B. anthracis* A16 | 1.332 | 1.213 | 1.308 |
| *B. cereus* AND 1313R | 0.223 | 0.218 | 0.135 |
| *B. cereus* F 3502/73R | 0.057 | 0.050 | 0.050 |
| *B. cereus* F4810/72 | 0.054 | 0.051 | 0.050 |
| *B. cereus* MADM 1279 | 0.061 | 0.050 | 0.053 |
| *B. cereus* AND 1315R | 0.058 | 0.054 | 0.051 |
| *B. cereus* NC 7401/2455 | 0.057 | 0.052 | 0.051 |
| *B. cereus* us HXM1 | 0.047 | 0.048 | 0.046 |
| *B. cereus* HXM2 | 0.057 | 0.061 | 0.053 |
| *B. thuringiensis* subsp. konkukian | 0.077 | 0.076 | 0.075 |
| *B. thuringiensis* subsp. iberica | 0.087 | 0.091 | 0.083 |
| *B. thuringiensis* subsp. pingluonsis | 0.054 | 0.051 | 0.051 |
| *B. thuringiensis* subsp. sylvestriensis | 0.123 | 0.225 | 0.137 |
| *B. thuringiensis* subsp. zhaodongensis | 0.053 | 0.048 | 0.052 |
| *B. thuringiensis* subsp. bolivia | 0.067 | 0.071 | 0.065 |
| *B. thuringiensis* subsp. graciosensis | 0.060 | 0.061 | 0.060 |
| *B. thuringiensis* subsp. pahangi | 0.059 | 0.060 | 0.058 |
| *B. thuringiensis* subsp. sinensis | 0.062 | 0.072 | 0.062 |
| *B. thuringiensis* subsp. tenebrionis | 0.130 | 0.130 | 0.117 |

**Table 2** **mAbs reactions with the vegetative cells of *B. thuringienesis* and *B. cereus* using sandwich ELISA**

| **Vegetative cells**  **(10^7^ CFU/ml)** | **OD_450 nm_** | | |
| --- | --- | --- | --- |
|  | **mAb 8G3** | **mAb 10C6** | **mAb 12F6** |
| *B. anthracis* A16 | 1.574 | 2.259 | 1.944 |
| *B. cereus* F 3502/73R | 0.060 | 0.060 | 0.105 |
| *B. cereus* F4810/72 | 0.022 | 0.030 | 0.022 |
| *B. cereus* MADM 1279 | 0.054 | 0.052 | 0.057 |
| *B. cereus* AND 1313R | 0.068 | 0.019 | 0.022 |
| *B. cereus* AND 1315R | 0.022 | 0.025 | 0.006 |
| *B. cereus* NC 7401/2455 | 0.023 | 0.026 | 0.014 |
| *B. cereus* us HXM1 | 0.026 | 0.047 | 0.073 |
| *B. cereus* HXM2 | 0.024 | 0.055 | 0.130 |
| *B. thuringiensis* subsp. konkukian | 0.020 | 0.010 | 0.035 |
| *B. thuringiensis* subsp. iberica | 0.045 | 0.057 | 0.087 |
| *B. thuringiensis* subsp. pingluonsis | 0.034 | 0.061 | 0.094 |
| *B. thuringiensis* subsp. sylvestriensis | 0.031 | 0.057 | 0.074 |
| *B. thuringiensis* subsp. zhaodongensis | 0.025 | 0.027 | 0.080 |
| *B. thuringiensis* subsp. bolivia | 0.039 | 0.026 | 0.051 |
| *B. thuringiensis* subsp. graciosensis | 0.036 | 0.079 | 0.017 |
| *B. thuringiensis* subsp. pahangi | 0.043 | 0.065 | 0.135 |
| *B. thuringiensis* subsp. sinensis | 0.034 | 0.031 | 0.080 |
| *B. thuringiensis* subsp. tenebrionis | 0.000 | 0.000 | 0.035 |
